# Attendant’s experience with the personalized citizen assistance for social participation (APIC)

**DOI:** 10.1186/s12877-020-01897-x

**Published:** 2020-11-25

**Authors:** Karine Gagnon, Mélanie Levasseur

**Affiliations:** 1 Faculty of Medicine and Health Sciences, Université de Sherbrooke, 3001, 12th, Avenue North, Sherbrooke, Quebec J1H 5N4 Canada; 2 Research Centre on Aging, Eastern Townships Integrated University Centre for Health & Social Services—Sherbrooke Hospital University Centre, 1036, Belvedere Street South, Sherbrooke, Quebec J1H 4C4 Canada

**Keywords:** Community participation, Individualized assistance, Aging, Community integration, Health promotion

## Abstract

**Background:**

To promote healthy aging, the social participation needs of older adults must be better met. Previous studies have shown the benefits of the Personalized citizen assistance for social participation (APIC), but few explored its influence on attendants. This study explored the assistance experience of attendants in providing the APIC to older adults with disabilities.

**Methods:**

A qualitative design inspired by a phenomenological approach was used with six female attendants who participated in individual interviews.

**Results:**

The APIC attendants felt useful, developed meaningful relationships with their older adults, and improved their self-knowledge. Attendants had the opportunity to reflect on their lives and self-aging. They contributed to older adults’ functional independence, motivation, and participation in social activities. Attendants encountered challenges related to withdrawn behavior in older adults, such as refusing to participate in activities.

**Conclusions:**

Considering the identified benefits of the APIC for attendants, further studies should explore personalized assistance to preserve older adults’ health.

## Background

Demographic changes lead to challenges requiring concrete interventions to preserve older adults’ health and functional independence while avoiding an overburdened healthcare system. In Quebec, some plans have been implemented to improve the living conditions of older adults and avoid or delay chronic illness and disabilities, such as the Quebec Action Plan for all ages [1]. Disability is defined as a reduction or a disturbance in the accomplishment of the individuals’ daily and social activities [2]. Despite these actions, some older adults’ social participation needs are still unmet [3, 4]. Defined as ‘*a person’s involvement in activities that provide interactions with others in the community*’ [5], social participation is a determinant of older adults’ health [6–8]. Indeed, social participation is a key element of well-being [9], quality of life [10, 11], and a strategy to preserve mobility [12]. Interventions to improve the social participation of older adults, whether they be individual, in a group, formal or informal, are often unavailable [13, 14] or respond inadequately to older adults’ needs [15]. When lacking social participation opportunities, older adults, especially those with disabilities, resign themselves to their situation and stay at home [3]. Many older adults are also reluctant to ask for additional help from their network in order to participate in social activities that could positively affect their health [3, 16]. Nevertheless, it is possible to develop, adapt, and, while meeting older adults’ needs, implement social participation interventions in the community. Among these interventions, a rather innovative one is the Personalized citizen assistance for social participation (APIC; in French: *Accompagnement-citoyen personnalisé d’intégration communautaire*), which has recently been adapted for older adults with disabilities [17].

### The personalized citizen assistance for social participation (APIC)

The APIC is a program comprised of weekly meetings of 3 h, conducted over a period of 6 to 18 months by trained attendants. During these meetings, the attendants encourage older adults to identify important but challenging social activities to accomplish in their community. The APIC was initially designed to support adults with traumatic brain injury (TBI) in their life plans. The APIC addressed the lack of resources in integrated health and social services centers [18]. As adults with TBI and older adults with disabilities are similar in terms of the personal or social restrictions limiting their social participation and their need for personalized assistance to carry out activities [19], the APIC was adapted accordingly. Results from this adaptation have shown that the older adults increased their functional independence, their sense of accomplishment, their satisfaction with social participation, and the frequency of their leisure activities after the APIC. The attendants allowed the older adults to share moments and accomplish social activities [17]. Although the personalized assistance in the APIC demonstrated an important role in guiding and supporting older adults through their projects to improve their independence and their satisfaction with their personal choices [20], little is known about personalized assistance and its benefits.

### Personalized assistance

According to Paul [20], assistance refers to “*both a function and a position, referring to a relationship and an approach that, to be specific, are none the less bound to adapt to each context and each relational matrix*” (p.13; free translation). Assistance is personalized when it is adapted to the needs of each individual. Personalized assistance is inexpensive and involves good use of community resources. It can foster social interaction and empowerment, and reduce the number of isolation cases in vulnerable populations [21]. Personalized assistance also promotes equality in relationships and reciprocal exchanges [22] in which attendants do not do something ‘for’ but rather ‘with’ someone, cooperatively and in partnership [23]. The aim of personalized assistance is to restore social relationships, and is particularly suitable for older adults who wish to remain socially active but have a limited social network. Assistance can be provided through projects, and can help the person acquire a higher level of functional independence, a better quality of life, and satisfaction with their personal choices [20].

To our knowledge, 24 articles have been published on social participation interventions with personalized assistance. Of these articles, six explored attendants’ experience. Using creative arts rather than the APIC, one article highlighted that, while helping isolated older adults, attendants experienced positive repercussions [24], but these were not specified. The five other articles are about the same attendants providing the APIC in 2009–2011 to adults with TBI and, instead of addressing an overall vision of the experience, focused specifically on its impact on the attendants’ mental health [25]. The majority of attendants reported personal benefits from their personalized assistance, such as the satisfaction of helping others and feeling useful when highlighting the abilities of adults with TBI [26], but these benefits were not detailed further. These articles emphasized that personalized assistance was a source of pleasure for attendants despite the absence of clear boundaries defining their role during the APIC [27]. To identify their personal limits and reduce ambiguous situations in their relationships with older adults, attendants expressed a need for coaching [28]. Attendants experienced several limitations, particularly related to the lack of motivation of the person they assisted during social activities, which was sometimes difficult to overcome [29]. To recruit and assist attendants in future, better knowledge of the attendants’ experience, and the resulting benefits, will be needed. Thus this study was aimed at exploring the attendants’ assistance experience in providing the APIC to older adults with disabilities.

## Methods

### Design

This study is part of a larger research program exploring the APIC’s impact on older adults with disabilities [17] and its feasibility [30], and used a qualitative method using a phenomenological approach. This approach helps describe a person’s experience, i.e. an attendant’s assistance experience as it happened, to understand its meaning and value [31]. Data were collected through interviews with attendants, logbooks completed during the APIC, and attendants meetings. This data underwent thematic content analysis using mix extraction grids [32]. The influence of the APIC on older adults’ quality of life, mobility habits, functional independence, social participation and leisure, as well as their experience with assistance 12 months after it ended, were also explored [33].

### Sampling

Of the ten attendants who previously participated in the APIC [17], six enrolled for the current study and gave their free and informed written consent. The other four attendants declined to participate, two of them expressing time limitations and the other two a lack of interest. This sample size achieved data saturation and favored in-depth exploration of the attendants’ assistance experience in the APIC. To be involved in the APIC, attendants had to speak French, have no criminal record, and provide adequate references. Recruited across several networks including *Emploi-Québec*, all attendants were non-professional citizens having previous experience with older adults, and were paid during the training, the APIC, and the meetings. The previous experiences of the attendants were mainly as family caregivers and volunteers within community organizations helping the older adults. One attendant also managed a retirement home.

### Intervention

The APIC was provided from November of 2013 to September of 2014 and involved weekly meetings of 3 h, conducted over a period of 6 months. To be eligible for the APIC, the older adults had to: (1) be aged ≥65; (2) live with moderate to severe loss of functional independence, based on a score ≥ 15 on the Functional Autonomy Measurement System (SMAF) [34]; (3) present normal cognitive functions based on a score ≥ 17 on the phone version of the Mini-Mental State Examination (ALFI-MMSE) [35]; (4) live in a conventional or residential home for independent or semi-independent older adults, and (5) communicate orally in French. The older adults had difficulty accomplishing at least one of their daily activities, for example, shopping, household chores and maintenance. The assisted older adults and the attendants were paired according to their interests and specific requests (e.g. paired with someone of the same gender). Before starting the APIC, each attendant underwent 2 days of training on aging, disability, community resources, and the personalized communication approach. During the training, the attendants also visited the day centre to be in contact with older adults with disabilities. With the help of presentations, videos and testimonials from older adults, the attendants developed their knowledge on, among other things, falls prevention and how to establish contact with older adults and further defined their role during the assistance. This training enabled the attendants to gain knowledge and develop tools to support the older adults in setting goals for significant social activities and encouraged their empowerment and community integration [30]. Throughout the APIC, the attendants encouraged the older adults to identify important goals related to their life plan and social activities carried out in the community. Such social activities could be going to the library and participating in the available activities at local community organizations. With the help of the attendants, the older adults who participated in the APIC gradually learned to mobilize their personal and environmental resources and meet their individual needs to feel as independent and satisfied as possible with their community integration. Although the purpose was not to enrol the older adults who have finished the APIC as future attendants, this participation has been seen in other version of the APIC [36]. During the APIC, the attendants completed a weekly logbook to document each older adult’s progression, and compile data about the session, such as objectives, activities carried out, and actions supporting the older adults in their life plan. The six attendants also completed logbook entries documenting their experiences with twelve older adults (see results) and attended four meetings. These meetings allowed the attendants to discuss the older adults’ progress and the challenges they faced, and if needed, to offer each other advice and support. The attendants were supervised by a management and partnership committee (MPC) comprised of the research assistant, the healthcare professionals, the study manager, the researchers, the individuals representing community organizations, the attendants, and the older adults. For this supervision, the MPC met every 4 months to review the logbooks, phone discussions, individual appointments and/or participation in attendants’ meetings.

### Data collection and tools

**Fig. 1:**
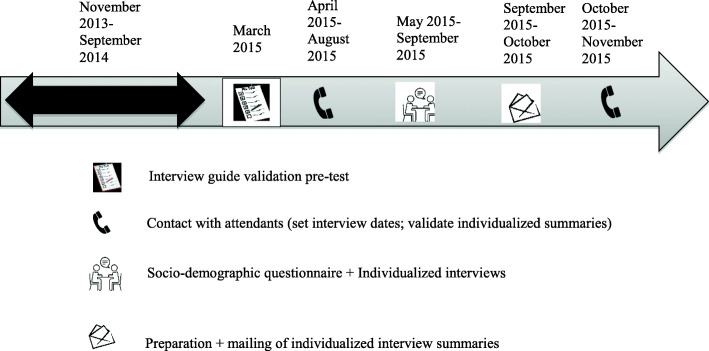
Data collection stages

Figure 1 illustrates the data collection stages. A semi-structured interview guide was used to allow attendants to revisit their assistance experience 12 months after the end of the APIC. The guide was based on the explicitation interview model [37], in which the interviewer intervenes as little as possible and creates conditions conducive to accessing participants’ prior knowledge or experience. The interview guide was validated by two qualitative research experts and was pretested with an attendant involved in a community organization. The guide included open-ended questions such as ‘*Tell me about your assistance experience*’ and ‘*What did you learn from your assistance experience?*’. About a year after the APIC, the attendants completed a socio-demographic questionnaire and participated in semi-structured individual interviews, lasting approximately 60 min. The interviews were conducted at the attendants’ homes, were digitally audiotaped, were transcribed, and were verified with respect to the wording used by the attendants. Following the interviews, the individualized summaries were prepared and mailed to the attendants, who were subsequently called to validate them. All attendants confirmed the compliance of their summaries. Moreover, to have a complete understanding of the attendants’ assistance experience, their logbooks as well as transcripts of their meetings were also collected.

### Data analysis

The sociodemographic characteristics of the attendants were described using medians and semi-interquartile intervals or frequencies and percentages, for numerical or categorical variables respectively. The attendants’ interviews, logbooks, and meetings underwent thematic content analysis using mix extraction grids [31] carried out using N’Vivo10 software. Emerging themes from the attendants’ interviews, logbooks, and meetings were organized and renamed in accordance with the Human Development Model - Disability Creation Process (HDM-DCP) [2]. This model allowed for a broader view of factors that could hinder or act as protective factors for social participation, and described maintenance of lifestyle habits (social participation). The HDM-DCP highlighted the importance of the attendants’ role in the APIC as an environmental determinant to improve older adults’ social participation. To enhance the reliability of the findings, one third of the data was coded by a researcher experienced in qualitative analysis.

## Results

The attendants who participated in this study were aged 56–61, [median ± semi-interquartile range (Md ± Q): 61.0 ± 2.0] and were all female (Table 1). The majority were single (2; 33.3%) divorced (1; 16.7%) or widowed (1; 16.7%). Half of the attendants reported incomes of $25,000 or more, and most were satisfied with their incomes [one was satisfied (16.7%) and three were very satisfied (50.0%)]. The attendants had either a college or a vocational diploma (*n* = 3; 50.0%) or a Bachelor’s degree (*n* = 3; 50.0%). Four attendants (66.7%) had previous volunteer experience prior to the APIC, and half of them (*n* = 3; 50.0%) had volunteered for more than 5 years. Half of the attendants assisted more than one older person during the APIC. Aged between 68 and 93 (Md: 82.0 ± 2.0), the majority of the older adults assisted by the attendants were women (*n* = 9; 75.0%), rated their health as good, but reported feeling depressed on occasion.

**Table 1:** Socio-demographic characteristics of attendants (*n* = 6)

Attendant #	Marital status^**a**^	Income^**b**^	Income satisfaction^**c**^	Education^**d**^	Volunteering involvement before the APIC^**e**^	Volunteering involvement (years)	Assisted older adults in the APIC
**A1**	1	4	1	1	1	12	4
**A2**	3	3	2	2	2	–	1
**A3**	3	1	3	2	1	5	1
**A4**	2	3	3	1	1	8	2
**A5**	4	2	2	2	1	4	3
**A6**	1	2	2	1	2	–	1

^a^ (1) Married / Common law; (2) Widowed; (3) Single; (4) Divorced / Separated

^b^ (1) < $15.000; (2) $15.001–$25.000; (3) $25.001–$40.000; (4) > $40.001

^c^ (1) Very satisfied; (2) Satisfied; (3) Dissatisfied

^d^ (1) College / Vocational; (2) Bachelor

^e^ (1) Yes; (2) No

Overall, all the attendants appreciated their experience during the APIC when assisting older adults in optimizing their social activities and life plan. The attendants’ assistance experience was described in terms of: 1) developing a meaningful relationship with older adults, 2) increasing their self-knowledge, 3) encountering challenges, and 4) acting as agents of change. Firstly, the attendants developed meaningful relationships with the older adults they assisted. All attendants reported they were caring with older adults, such as being generous with their time and help, or worrying about the older adults’ health, as one attendant indicated: “*I told her that I was really happy to see that she was much better and looked good [after an illness episode]. I felt she was much less anxious and leading a fuller life*.” (Logbook of A3; LA3). The attendants said their relationships with their older adults were pleasant, based on trust, and allowed for the sharing of common interests: “*I was so happy to share with him one of his passions, his love of books. A moment of pure happiness*.” (LA1). The attendants reported being good listeners, which allowed the older adults to confide in them and express their emotions. Moreover, the attendants mentioned that their presence reassured the older adults, gave them energy, cheered them up, and helped them maintain social ties in the community.

Secondly, the APIC contributed to the attendants’ self-development, especially when they had to be assertive because of the older adults’ attitudes, such as when they refused to look at the social activities being carried out in the community (*n* = 7) or lacked interest in participating in them (*n* = 3). The attendants’ self-assertion helped develop an egalitarian relationship, in which the older adults understood more about the attendants’ role and acted more respectfully towards them, as illustrated in the following example:

“*I found her very stiff, she did not want to make a connection and just saw me as an employee [ … ]. At one point, I had to sit down with her and tell her: ‘I’m not comfortable with that’. I think that helped and eventually she realized that, it’s true, she’s not my employee*.” (A5).

One third (*n* = 2) of the attendants reported that the APIC gave them an opportunity to increase their self-knowledge, their self-esteem, and their self-satisfaction. They recognized their own qualities and abilities. They realized that they could influence those close to them with their advice, including the assisted older adults, which increased their self-esteem. Through the APIC, the attendants (*n* = 4) initiated contemplation of the aging process for themselves as well as for the older adults around them, and adapted their vision of the aging process to the assisted older adults’ reality. More precisely, these adaptations related to life in a residence and the care services provided by the public health system. Their experiences also allowed half of the attendants to reflect on, and ask questions about, their responsibilities towards assisted older adults and the difficulties associated with unclear boundaries with them. To maintain a positive and appropriate relationship with older adults, four attendants needed to distance themselves from the older adults’ personal problems or important decisions to be made. Distancing themselves was not always easy for the attendants, as mentioned by one who assisted a blind older adult experiencing a couple crisis during the APIC: “*The hardest thing was to be able to help him without interfering in decisions [to split up with his wife and move into another apartment], to keep myself at a distance from that*.” (A1 in meeting; MA1). Such a situation was beyond the role of the attendants and required the care of a health professional, as explained by the same attendant: “*I felt overwhelmed when the couple broke up [resulting in an interruption of the APIC with the attendant for two weeks]. I would have liked the social worker to act a little faster, come and support them during the crisis because there was violence*.” (A1). A third (*n* = 2) of the attendants reported experiencing situations beyond their role.

Thirdly, the attendants faced some challenges related to the older adults. The physical condition of some older adults restricted opportunities for, or the duration of, social activities on several occasions. The health of some older adults (*n* = 6) deteriorated during the APIC and prevented them from participating in social activities in the community, leaving most of the attendants (*n* = 4) to run out of ideas about social activities that could be done with them, as expressed by one attendant: “*[The older woman I assisted] fell in her apartment and, from that moment, she was not able to walk and had no energy. I did not know what to do with her [to help her carry out social activities].*” (A4). Some older adults exhibited inappropriate behavior (*n* = 7) that at times compromised the interpersonal relationships with the attendants or participation in the social activities. On occasion, some attendants (*n* = 2) were yelled at, heard derogatory words, or felt impatience towards them from the older adults, as reported by one attendant who was faced with such a situation: “*I have difficulty with screams, anger and explosive frustrations [ …*]. *It was natural for [the older woman I assisted] to use a very dry tone and it felt aggressive.*” (LA5). Some attendants (*n* = 3) reported that some older adults (*n* = 4) had rigid personalities, such as demonstrating a closed attitude or refusing to pay for activities, which limited social exchange opportunities. Some attendants (*n* = 2) were concerned about the older adults’ physical abilities because of their refusal to use appropriate technical assistance during the APIC, as illustrated by one attendant: “*She had difficulty walking, but refused to use her walker.[ …*] *I stayed close by, but it worried me the whole time*.” (LA6).

The attendants (*n* = 5) reported that another challenge during the APIC was the older adults’ refusal to get involved in social activities in their community, whether for lack of interest in doing activities with peers or other needs deemed more meaningful for them, such as chatting with the attendants. Talking with the older adults about the goals of social activities then became difficult due to this lack of interest, as experienced by this attendant: “*Joining [community] organizations was often discussed with her [older adult], but she really did not want to. She said: ‘No, I do not need that’*.” (A6). On some occasions, seven older adults expressed needs other than doing social activities outside their homes (i.e. watching television). Likewise, three older adults did not want to maintain social relations with others in the neighborhood, especially because of their uncertainty about socializing with peers and fear of being rejected or denigrated when joining an existing social group. Furthermore, the attendants reported that some older adults (*n* = 3) refused to compromise on, for example, paying for some social activities or calling for adapted transportation to attend a community activity, making social integration difficult, as was the case for this older adult: “*We could have gone out every week, but she knew there were fees if we ever went out. She didn’t want to pay for taxis, she said it was too expensive, and she didn’t want to take adapted transport because she said it was lousy, it wasn’t made for her, so we stayed at home*.” (LA5).

Fourthly, the attendants mentioned acting as agents of change during the APIC. All of the attendants reported having identified opportunities for the older adults to get involved in the community, which contributed to their social participation. Every attendant also reinforced empowerment in older adults by maintaining their functional independence, encouraging them to try new social activities, and accomplishing activities outside their home. This example illustrates how the attendants positively contributed to the older adults’ empowerment, including their ability to make choices independently, as described by one attendant: “*Madam is increasingly gaining confidence regarding our sessions. She made her choice without waiting for my response [to undertake social activities]*.” (LA5). Although it fell outside their role, all attendants felt that they had contributed to social and leisure tasks in the older adults’ lives, such as managing mail and phone calls, which were sometimes stressful and where assistance from attendants was precious and helpful: “*She mentioned that the afternoon had passed too quickly but that she was very happy with the calls [to resolve a problem with the older adult’s unpaid bills] that I had made for her, because she felt reassured*.” (LA6). Moreover, attendants (*n* = 3) reported having enhanced the older adults’ qualities and abilities, by emphasizing progress and congratulating them on initiatives, which subsequently encouraged the older adults to take action. One attendant observed changes in the assisted older adult’s attitude and motivation towards opportunities for social activities after pointing out her social and physical abilities: “[During the APIC] *She really opened up to people, to the world, there were no more barriers. This project enabled her to take small outings, to chat, to say you can do it, to restore the person’s confidence, which made all the difference*.” (MA1). Finally, some attendants (*n* = 4) said that they contributed to the older adults’ social participation by encouraging them to integrate into the community, as expressed by one attendant: “*I suggested that she sign up for creative workshops. Following this suggestion she informed me that she had just enrolled in Tai Chi at her community center*.” (LA5). The attendants (*n* = 2) supported the older adults in making permanent changes with regard to their social participation that was better adapted to their needs and their abilities, and in making long-term commitments in their community. At the end of the APIC, all the attendants wanted to maintain the friendship bonds with the older adults. Half (*n* = 3; 50%) of the attendants continued contact and visits with the older adults for a few months after the end of the APIC, but no longer maintained relationships with them 12 months later. The other half of the attendants are still visiting or hearing from the older adults because they are attached to them and they still have a place in their lives, as reported by one attendant: “*I have a lot in common with [the older woman I assisted], what she likes are things I like too. So, for sure, we continue to see each other*.” (A1).

## Discussion

The aim of this study was to explore the attendants’ assistance experience when providing the APIC to older adults with disabilities. During the APIC, the attendants developed meaningful relationships with the older adults. The APIC allowed the attendants to increase their self-knowledge and reflect on their own aging and that of the older adults around them. The attendants also faced challenges, such as those related to personality and behavior of the older adults, and performed several roles, including being agents of change and contributing to the older adults’ social participation. To our knowledge, few studies have described the benefits to attendants of providing personalized assistance to vulnerable populations such as frail older adults.

The attendants developed meaningful relationships throughout the personalized assistance process, which, for three of them, continued even after the APIC had ended. As also observed by Stevens, Barlow & Llife [38], the development of strong relationships during a 24-week physical activity program was an important aspect of personalized assistance, leading to enjoyable times and friendships lasting beyond the length of the program. During the APIC, the attendants were generous with their time, and were concerned about the health of the older adults and their social ties within the community. These findings are similar to those of the study by Outcalt [39], where paid companions provided assisted older adults with emotional and social support through home visits, and kept them engaged in the community.

The attendants increased their own self-knowledge during the APIC. Moreover, as outlined in the current study and the one by Therriault & Samuelson [26], they increased their self-esteem and self-satisfaction. Through the personalized assistance process, the attendants felt useful [28] and valued by the older adults [26]. The lack of defined boundaries for assistance affected the attendants’ assistance experience. They had difficulties in clearly establishing what was expected from them in the older adults’ life plans. This absence of clear boundaries was also reported by attendants who participated in another study [29]. This confusion over boundaries and the desire to better understand and define the attendants’ role led Therriault and colleagues to make the following five recommendations: 1) support the attendants more closely in establishing the right amount of distance in their relationships; 2) consider all the players in the process (i.e. family, community, etc.); 3) make the assisted person and his desires the focus of the personalized assistance; 4) acknowledge that an attendant will not know everything; and 5) recognize the possibility of being transformed throughout personalized assistance [29]. These recommendations could provide greater understanding of what is expected of attendants during personalized assistance and could define more clearly their roles with older adults in the future training of attendants. According to the findings from this current study, the recommendations by Therriault & colleagues [29] remain valid, and the quotes from several attendants support their relevance. For example, some of these quotes reveal the attendants’ difficulties in establishing boundaries within their relationships with the older adults, and their important role as an environmental determinant and support in the social participation of the older adults.

In accordance with the challenges mentioned in the current study, Therriault and colleagues [25, 29] reported similar assistance experiences by attendants regarding certain personality or behavioral traits in older adults, their lack of motivation, and their refusal to participate in social activities, all of which compromised their peer relationships or their social integration. According to other studies [40, 41] and the current findings, these challenges could be related to a fear of being judged by peers. As described in the current study, and also identified by Therriault and colleagues [28], the attitude or behavior of some older adults that hindered their relationships with others, or represented an obstacle to subsequent activities and resulted in uncomfortable situations for the attendants.

Finally, as outlined in the current findings on attendants acting as agents of change, personalized assistance contributed to functional independence [20], motivation, and long-term commitment [42] in older adults. The attendants contributed to the older adults’ empowerment when promoting opportunities for them in social activities. The contribution by the attendants to assisted older adults’ social activities was as discussed in the study of Wilson, Bigby, Stancliffe, Balandin, Craig & Andersson [43]. Attendants supported adults with intellectual disabilities in their social participation and helped them carry out significant activities in the community. Attendants allowed adults to join, participate, and be included in significant social activities. As community leaders, attendants ensured that adults were included in activities and conversations, which was a central aspect to support their social participation [43].

To further develop knowledge on personalized assistance, the implementation of the APIC in one or more community organizations should be studied. The missions of those organizations are to overcome isolation and encourage social contacts in older adults. Moreover, future studies should include a larger sample and use a robust design such as randomized clinical trial [44]. Since the APIC involves non-professional volunteers-citizens, the costs of the training, supervision and follow-up should also be documented. Unlike the peer health navigators [45], the attendants who participated in the APIC came from a local community and did not have a specialized health training to empower older adults and provide them with individualized services and support. Moreover, in the APIC, the attendants assisted older adults in their social participation needs, which differ from the peer health navigators, who were more likely to be health care professionals intervening according to specific health care needs and compensating for fragmented health care delivery. In the APIC, the attendants’ lack of training and recognized professional experience might increase the burden of community organizations in managing the intervention and complexify the sustainability of its implementation in community settings [36]. Since the APIC optimizes community resources to promote the health and independence of older adults and might reduce the pressure on the public health care system, governmental help is required to support the implementation of the APIC. Such help might involve the funding of community organizations through sustainable public funds. By fostering economic and social support to community organizations, coordinated care and access to health services might be increased and the health and quality of life of the older adults with disabilities be maintained. Likewise, the benevolent communities must also be further promoted and supported by policies, for example with the help of the Quebec Action Plan for all ages [1]. Finally, governmental support should also help with funding research on the costs and feasibility of the APIC in local communities and involving technologies as well as peers and young adults.

### Study strengths and limitations

This study is the first providing an in-depth exploration of the attendants’ assistance experience in the APIC with older adults with disabilities. Interviews with the attendants were audio recorded, transcribed, and verified, which increased the reliability of the data collected. One third of the qualitative data was co-coded with the thesis supervisor, which also improved the reliability of the findings. Since the APIC was completed 12 months before interviews with the attendants, the effects of time may have altered their recall of the assistance experience. In order to minimize the effects of time, certain strategies were deployed, such as the explicitation interview. Moreover, social desirability on the part of the attendants could be a potential bias, even if they were informed about the absence of right or wrong answers. As with other qualitative studies, the findings from our study may have been influenced by the researchers and are sensitive to context.

## Conclusions

This study explored the attendants’ assistance experience when providing the APIC to older adults with disabilities. In the current study, the APIC was a series of weekly 3-h sessions over 6 months, carried out by trained attendants. The APIC showed several benefits for the attendants, including the development of meaningful relationships with the older adults and an increase in their self-knowledge. The attendants also encountered challenges during the APIC, such as the older adults’ physical conditions and their refusal or their lack of interest in participating in social activities. Moreover, the attendants acted as agents of change for the older adults, such as reinforcing empowerment and enhancing qualities and abilities. Although, in the current study, none of the older adults who had finished the APIC continued as an attendant, another study observed such involvement [36]. Up to now, personalized assistance has not often been described in the literature, but the current findings of this study have shown several benefits. Personalized assistance can meet the challenges of aging and help optimize community organizations services according to the needs of older adults. Further studies will be needed to document the positive effects of the APIC for attendants.

## Data Availability

Data are available upon request to corresponding author.

## Abbreviation

APIC: Personalized citizen assistance for social participation (in French: *Accompagnement-citoyen Personnalisé d’Intégration Communautaire*)
